# Optimization of process variables for acetoin production in a bioreactor using Taguchi orthogonal array design

**DOI:** 10.1016/j.heliyon.2020.e05103

**Published:** 2020-10-05

**Authors:** Abiola Ezekiel Taiwo, Tafirenyika Nyamayaro Madzimbamuto, Tunde Victor Ojumu

**Affiliations:** Department of Chemical Engineering, Cape Peninsula University of Technology, P.O Box 1609, Bellville, 7535, South Africa

**Keywords:** Bioengineering, Biotechnology, Microbiology, Acetoin, Fermentation, Optimization, Taguchi design

## Abstract

Microbial production of acetoin is eco-friendly and inexpensive when compared with its synthetic methods of production. In the present findings, bioproduction of acetoin in a typical bioreactor was discussed with a view to ascertain the seemingly comparative advantage of bioreactor system over shake flask, and more importantly, to confirm that corn steep liquor can indeed adequately be used as a replacement for other organic nitrogen sources. Taguchi design was statistically used to optimized the fermentation process which resulted in a 3-fold increase in molar yield (83%) corresponding to a six-fold increase in acetoin concentration (63.43 g/L), as compared to a similar study conducted in a shake flask. Although agitation rate was observed to be the most controlling, the bioreactor may underperform at agitation rate greater than 300 rpm. The optimum parameters for acetoin production in this study were 300 rpm agitation, 1.5 slpm aeration, 2 days fermentation time, and pH 6.5. The results show that the commercial production of acetoin can be envisioned using a biological approach that may be of economic advantage.

## 1 Introduction

Flavour products have turned into inevitable supplements used in food industries (Teixeira et al., 2002). This is such that it keeps the taste of food, pharmaceutical, and cosmetic products attractive to consumers thereby increasing the market share of such products by giving it a competitive advantage. Currently, the paradigm shift toward the consumption of natural or biological products rather than synthetic counterpart is placing a huge challenge on the commercial availability of these products. For example, acetyl methyl carbinol also known as acetoin is a yellowish liquid that is often utilized as flavour enhancement and sweetener in the production of dairy products, coffee, butter and milk, and other chemicals and pharmaceutical products (Effendi et al., 2013). Production of acetoin have been reported using different chemicals for its synthesis, however, alternative pathway using biological approach acetoin production has been reported (Zhang et al., 2016). Microbial production of acetoin is known to be nature friendly and inexpensive when compared with petroleum-derived methods of production (Liu et al., 2011). It has been shown that the biological production of acetoin is influenced by operating parameters such as pH, aeration, carbon sources, agitation, nitrogen sources, temperature and fermentation time (Tian et al., 2014; Taiwo et al., 2018). While Tian et al. (2014) reported an optimum yield of 46 g/L acetoin from glucose, we have shown that corn steep liquor can supply all the microbial nitrogen requirements without the addition of yeast extract. This approach may further reduce the production cost especially if commercial production is envisaged (Taiwo et al., 2018). Although most of the effects of the controlled parameters have been tested and established using relevant optimisation technique such as response surface methodology (Tian et al., 2014), they are mostly limited to shake flask studies. However, the challenge is the development of a scale-up process for commercial production of acetoin. Current studies on acetoin production in the bioreactor system are promising, fermentation process is more effective in the bioreactor system compared to the shake flask due to the fact that the bioreactor offers control of some process variables as such to promote optimum microbial performance. The superior performance of bioreactor studies compared with shake flask can be attributed to the effective growth environment provided within bioreactor systems. Variables such as dissolved oxygen concentration, pH, foaming level, agitation, temperature, gas mixture (nitrogen, oxygen, air, carbon dioxide) can be controlled in a bioreactor system, thereby giving it a comparative advantage over a shake flask system (Obom et al., 2013). Some of the shortcomings in shake flask studies do not apply to bioreactor studies.

It has been shown that improved yield of acetoin can be achieved in bioreactor operation using different strategies through genetic engineering, such as *Bacillus subtilis* and *Saccharomyces cerevisiae* encoding (Xiao et al., 2007; Bae et al., 2016, Tian et al., 2016), and isolating *Bacillus* species (Xiao et al., 2007). Although improved acetoin was reported by Tian et al. (2014) using a mixture of organic nitrogen sources, it was shown that corn steep liquor could supply the necessary ingredients required for optimum microbial performance (Taiwo et al., 2018). In addition to the above, the process optimisation approach using statistical tools has been used quite often to predict optimum process parameters. Experimental design using the Taguchi method is of interest as it reduces the number of experiments significantly while still achieving remarkable outputs, characterised by better process performance and stability of the experimental design (Chen et al., 2017). Taguchi design defined the significance of statistically aligned experiments in predicting the yield on various factors used in the experimental design (Das et al., 2014). The Taguchi design method makes use of fractional factorial and is referred to as orthogonal arrays (OAs). This helps in rationalizing multiple process variables while reducing the total number of experiments conducted. The choice of an appropriate OA is a function of the number of control factors and their numerical levels (Shahavi et al., 2016), the theoretical details can be found elsewhere (Dar and Anuradha, 2018).

The present study investigated the bioproduction of acetoin in a typical bioreactor a view to ascertain the seemingly comparative advantage of bioreactor system over shake flask, and to confirm that corn steep liquor can indeed adequately be used as a replacement for other organic nitrogen sources. More importantly, the process was statistically optimized by conducting a fewer number of experiments using Taguchi design to improve the yield parameter of acetoin in the bioreactor system.

## 2 Materials and methods

### 2.1 Microorganism and inoculum preparation

*B. subtilis* CICC 10025 was preserved on agar slants using this highlighted media (g/L): glucose 10, beef extract 10, peptone 10, sodium chloride 5, and agar 16 at pH 7.0. A starter culture was prepared by growing the bacterium in 50 mL of the following media in a 250 mL shake flask for 10 h with agitation of 150 rpm and temperature at 37 °C: glucose 60 g/L, beef extract 10 g/L, peptone 10 g/L yeast extract 10 g/L and sodium chloride 5 g/L at pH 7.0 (Xiao et al., 2007).

### 2.2 Fermentation medium

The fermentation medium for this study was optimized in a shake flask experiment in our previous study (Taiwo et al., 2018). (g/L): glucose 78.40, K_2_HPO_4_ 0.5 g, CH_3_COONa 0.5 g, NaCl 5 g, and MgSO_4_.7H_2_O 0.5 g, corn steep liquor 15 g. The pH of the medium was adjusted as specified in the Taguchi design and was sterilized in an autoclave at 121 °C for 15 min (Taiwo et al., 2018).

### 2.3 The setup of bioreactor

**Figure 1:**
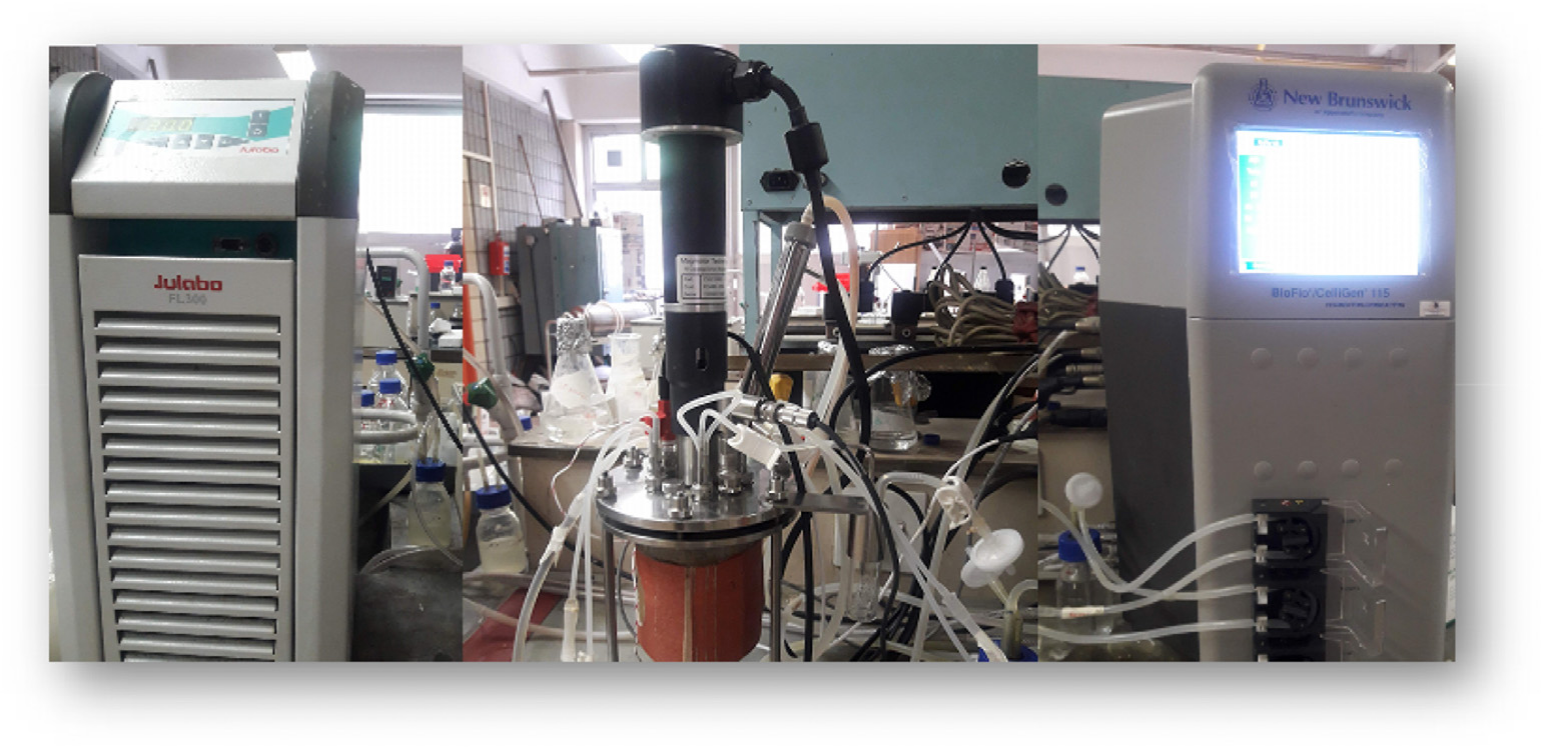
Experimental setup of bioreactor.

A 1.3 L laboratory-scale fermentor (BioFlo/CelliGen 115, New Brunswick Scientific Co., and Eppendorf, Germany) was used for acetoin scale-up study. The fermentor was connected with a driven stirrer at the top and two six-blade Rushton impellers attached to the agitation drive shaft (diameter 52 mm, width 16mm, length 18mm). The theory of design of a typical bioreactor has been discussed in detail elsewhere (Doran, 2012). The bioreactor was fitted with a 316 stainless steel sparger to ensure gas bubble dispersion and provide a high rate of oxygen transfer within the vessel. The maximum allowable levels of agitation and aeration were 1200 revolution per minute (rpm) and 5 standard litres per minute (slpm). A two-point standard calibration method (pH 4 and 7 buffers) was used to calibrate the pH electrode. The pH of the medium was stabilized with a measurement error of 0.01 by automatic addition of base (2 M NaOH) or acid (1N HCL). The bioreactor control station was connected directly with a recirculating chiller (FL300) that helped to cool the condenser and maintain temperature stability. The temperature of the bioreactor was set at 37 °C while other operating conditions (agitation, aeration, time, and pH) were sets as designed by the experiment carried out. Seed culture was first grown in a 250 mL flask with 100 mL cell suspension volume for 24 h at a temperature of 37 °C. The inoculation in the bioreactor was done with a bacterial seed culture of 3% v/v inoculum size. The sterilization of the bioreactor was done prior to the inoculation with the seed culture. Biocommand software (New Brunswick Scientific) was used for monitoring the bioprocess and data collection in the bioreactor (Seifan et al., 2017). The actual bioreactor set-up is displayed in Figure 1.

### 2.4 Taguchi orthogonal array design

Taguchi L9 orthogonal array (OA) design was used in this experiment. Agitation, aeration, pH, and fermentation time were used as variables and each factor was set at three levels, as shown in Table 1. In Taguchi L9 OA, a total number of 9 experiment run is expected to be carried out. This method provides an easy, effective, and structured approach to ascertain the most optimal process variables (Shahavi et al., 2016). The experiment layouts in Taguchi methods with L9 array for acetoin scale-up study are shown in Table 2.

**Table 1:** Selected process variables and respective levels in the experimental design.

	Process variables	Units	Level 1 (L_1_)	Level 2 (L_2_)	Level 3 (L_3_)
(A)	Agitation	rpm	150	300	400
(B)	Aeration rate	slpm	0.5	1.0	1.5
(C)	Time	days	2	4	5
(D)	pH	-	Uncontrolled	6.5	7.0

∗rpm; revolution per minute, slpm; standard litre per minute (unit of volumetric flow of gas and a function of total volume per batch).

**Table 2:** Experimental layout for acetoin batch study in coded form.

Batch runs	Agitation (rpm)	Aeration rate (slpm)	Time (days)	pH
1	1	3	3	3
2	2	2	3	1
3	3	2	1	3
4	2	3	1	2
5	2	1	2	3
6	1	1	1	1
7	1	2	2	2
8	3	1	3	2
9	3	3	2	1

#### 2.4.1 Analysis of the Taguchi orthogonal array experiments

The Minitab statistical software (version 17) was employed for the analysis of the experimental data obtained from the fermentation experiment. The objective function for the optimization studies in Taguchi design is obtained from the signal-to-noise ratio (S/N), usually the logarithmic function of the expected output. For every experimental run, signal to noise (S/N) ratio is equivalent to the-larger-the-better objective function as computed using Eq. (1) expressed below:

(1) $$\frac{S}{N}=-10\;\log\left(\frac{1}{n}\sum_{i=1}^{n}\frac{1}{{y_{i}}^{2}}\right)$$

The terms in Eq. (1) are defined as; *S/N*, the signal to noise ratio; n, the number of experiments carried out; *i* is the experimental run number, and y is the output result of the experiment.

### 2.5 Acetoin concentration determination

The concentration of acetoin was determined using the modified Voges-Proskauer test reaction (Speckman and Collins, 1982). A known volume of acetoin broth was pipetted into a 25 mL calibrated flask. A total of 2.5 mL of 1-naphthol solution (4 g of Naphthol dissolved 1n 100 mL isopropyl alcohol) and 1 mL of creatine solution (0.5 g creatine dissolved in 1 M NaOH solution) were added. A vortex mixer was used to shake the mixture vigorously and maintained at 30 °C as described by Campo and Carmenálajo (1992). A UV–Visible Spectrometer (2020 GBC Cintra model) was used to prepare a standard curve that estimates absorbance against the concentration of the solution. The absorbance for the solution was recorded after 40 min at 530 nm.

## 3 Results and discussion

### 3.1 Shake flask optimization study

The optimized process conditions for acetoin production in an incubatory shake flask study have been reported (Taiwo et al., 2018). It was shown that 10.70 g/L of acetoin can be obtained in a shake flask experiment using Box-Behnken design in a response surface methodology which reveal 78.40 g/L, 15% w/v, and 2.70% v/v as optimum parameters for glucose concentration, corn steep liquor, and inoculum size as respectively. These conditions were used as the basis for the bioreactor production study of acetoin.

### 3.2 Effects of aeration and agitation on acetoin production in the bioreactor

**Figure 2:**
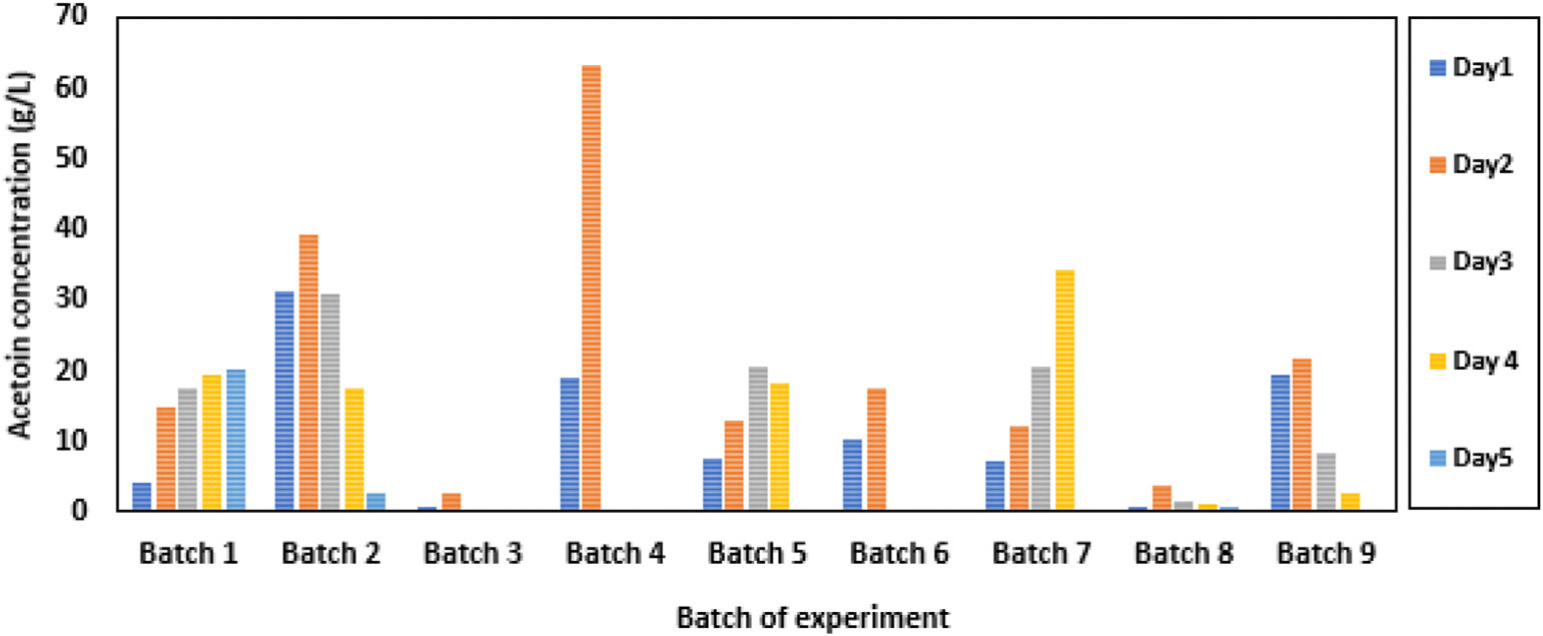
Batch production of acetoin production showing the effects of aeration, agitation, and pH.

The production of acetoin was studied at aeration rates of 0.5, 1.0 and 1.5 slpm while the agitation speed of the impeller was varied between 150-400 rpm at a temperature of 37 °C. The results show that high agitation of 400 rpm was not favourable for acetoin production when aerated from 0.5 -1.0 slpm (Figure 2). This was due to excessive foaming at these conditions. Foam has been reported to be a disadvantage in some fermentation processes as it may deprive microbes of the required nutrients. It has been reported that the formation of foam in bioprocess could be attributed to hydrodynamic conditions resulting from a gas introduction (aeration), composition of the medium, cell growth, metabolite formation, and the geometry of the bioreactor (Vardar-Sukan, 1992). Increasing the flow rate of air coupled with foam-stabilizing nutrients and other complex medium presents in the broth, make fermentation processes prone to foaming (Junker, 2007).

A maximum of about 2.57 and 3.69 g/L of acetoin was achieved at day two of this study as shown in batch 3 and 8 respectively. However, at a reduced agitation rate of 300 rpm (batch 4 and 5) and with aeration increase from 0.5 to 1.5 slpm, maximum acetoin concentration of 63.43 and 20.46 g/L were obtained on day two and three of fermentation respectively. While an increase in agitation and aeration are key drivers in the determination of the total mass and oxygen transfer in fermentation processes (Bajaj and Singhal, 2010), the authors further stressed that agitation plays the role in the movement of nutrients and flow of air and oxygen, while aeration supplies oxygen to the culture medium and support in the mixture of the fermentation broth, especially when the agitating speed is very slow. This study showed that agitation beyond 300 rpm may be counterproductive for acetoin production. Although it may be expected that the extended fermentation period allotted to the lower aeration rate (batch 5) would give a result similar to batch 5, the concurrent increase in the pH might be responsible for the observed reduction of acetoin concentration.

### 3.3 Effects of pH (controlled and uncontrolled) on acetoin batch production

Figure 2 also shows the effect of pH under the controlled condition on acetoin production (batch 1, 4, and 5). Although it has been reported that Bacillus strains could function in a fermentation medium at low pH range and that for acetoin production pH range 6.5–7.0 is recommended (Dai et al., 2015), this study shows that the microbe may underperform from pH 7 and beyond as shown in data from batch 4 and 5 when compared at day 2 period of fermentation. It can further be inferred from the batch 1 experiment, however, that the microbe may indeed underperform at this pH. It can be observed further that in an uncontrolled pH (batch 2) experiment, acetoin production continues to increase to a maximum at day 2 when the pH was approaching or at 7, and then declined steadily until the fermentation was terminated on day 5 at solution pH of 7.45. A similar trend may be expected for batch 6. In all the controlled pH experiments, batch 1,3, and 5, the microbe underperformed as the pH was controlled at pH 7 ± 0.1, batch 3 was particularly limited due to unfavourable agitation rate parameter.

### 3.4 Signal to noise ratio

Taguchi design uses the signal to noise (S/N) ratio to assess the value of significance in the choice of a variable when experiments are performed. S/N is characterized into three groups: nominal is better, the-smaller-the-better, and the-larger-the-better. For this study, the-larger-the-better was used to achieve a high yield of acetoin in the batch fermentation study. Therefore, as the delta value of S/N increases, the better the output yield. The optimum variables in this approach were obtained by sorting the delta value in increasing order of significance. A higher delta value indicates a significant effect of the variable on the whole experimental study.

**Figure 3:**
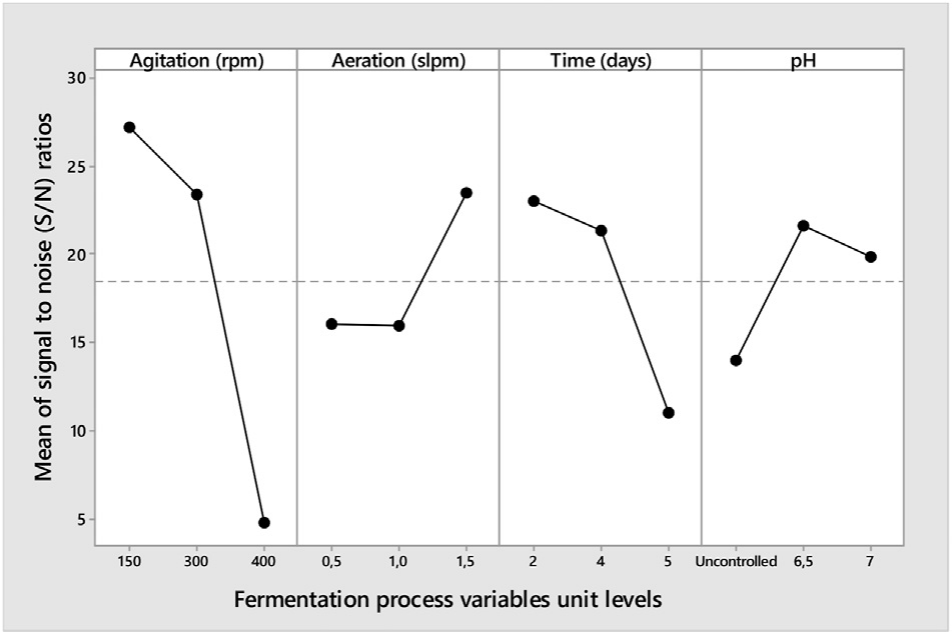
Main effects plot for S/N ratios and the corresponding fermentation process variables.

Based on Taguchi, variability of the resulting yield with reference to noise factors should be at a minimum while variability with respect to signal factors should be maximized. Table 3 presents the outcome of S/N ratio analysis against the fermentation variables and acetoin concentration (g/L). In Table 4, S/N ratio analysis revealed that the optimum condition for acetoin production was similar to that obtained using main effects plot (300 rpm, 1.5 slpm, 2 days, and pH 6.5) of the fermentation variable. The results show that agitation was the highest influencing parameter in improving acetoin yield (Figure 3).

**Table 3:** Acetoin production variables, concentration, and S/N ratio.

Batch number	Agitation (rpm) (A)	Aeration rate (slpm) (B)	Time (days) (C)	pH (D)	Acetoin conc. (g/L)	S/N ratio
1	150	1.5	5	7.0	20.32	26.16
2	300	1.0	5	Uncontrolled	2.81	8.97
3	400	1.0	2	7.0	2.57	8.20
4	300	1.5	2	6.5	63.43	36.05
5	300	0.5	4	7.0	18.14	25.17
6	150	0.5	2	Uncontrolled	17.39	24.81
7	150	1.0	4	6.5	34.45	30.74
8	400	0.5	5	6.5	0.80	-1.94
9	400	1.5	4	Uncontrolled	2.57	8.20

**Table 4:** Response table for the signal to noise ratio.

Level	Agitation (A)	Aeration (B)	Time (C)	pH (D)
Units	rpm	slpm	days	-
1	27.24	16.01	23.02	13.40
2	23.40	15.97	21.37	21.62
3	4.82	23.47	11.07	19.84
Delta	22.42	7.50	11.95	7.62
Rank	1	4	2	3
Optimum	A1	B3	C1	D2

The peak of the plots of each variable was chosen as depicted in Figure 3, as evidence that the ratio was the-larger-the-better, a term used for optimization in Taguchi orthogonal design. Similarly, optimum conditions can be observed in Table 4 by the delta values. Consequently, the optimum conditions obtained by this approach were as follows: A1, B3, C1, and D2.

### 3.5 Analysis of mean (ANOM)

**Figure 4:**
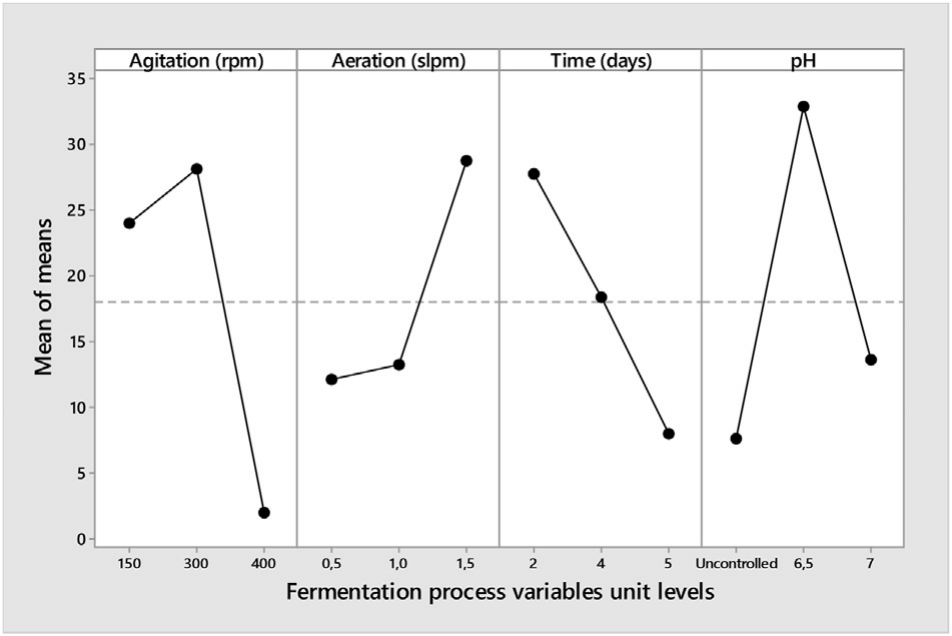
Main effects plot for the mean of fermentation variables.

Table 5 shows a response for analysis of mean (ANOM) and it demonstrates the equality of sample means. The primary focus of the ANOM is to test the effects of a designed experiment in which all the variables were fixed (Dar and Anuradha, 2018). The ANOM in Table 5 is estimated using the Eq. (2) below:

(2) $$m=\frac{1}{9}\sum_{j=1}^{9}\beta_{j}$$

where j represents the number of experimental runs from 1 to 9; m is the overall mean level result; β is the dependent variables (acetoin concentration). In Table 5, A, B, C, D were independent fermentation variables, the selected bold numbers were the minimum in every column of the three-step level, as per range, and used in setting the rank for all the variables. Based on the main effects plot for a mean of fermentation variables as shown in Figure 4, the optimum combination was agitation of 300 rpm, aeration of 1.5 slpm, fermentation time of 2 days, and pH of 6.5. The same optimum was coded as A2, B3, C1, and D2 in Table 5.

**Table 5:** ANOM response table for means.

Level	Agitation	Aeration rate	Time	pH
	A	B	C	D
Units	rpm	slpm	days	-
1	24.05	**12.11**	27.80	**7.59**
2	28.13	13.28	18.39	32.89
**3**	**1.98**	28.78	**7.98**	13.68
Delta	26.15	16.67	19.82	25.30
Rank	1	4	3	2
Optimum	A2	B3	C1	D2

Delta = maximum level of the mean - a minimum level of mean. The bold numbers indicate the minimum level of the means for each fermentation parameters.

### 3.6 Analysis of variance (ANOVA)

Analysis of variance (ANOVA) is used to examine the relationship between the yielded output and input variables of an experiment. In Table 6, ANOVA was used to measure the contribution of each fermentation variable in the batch study of acetoin.

**Table 6:** Analysis of Variance for Means and contribution of fermentation variables.

Variable	Units	Degree of freedom	Adj SS	Adj MS	Percentage contribution	Rank
Agitation	rpm	2	1187.47	593.74	35.52	1
pH	-	2	1046.59	523.29	31.31	2
Time	days	2	589.75	294.87	17.64	3
Aeration	-	2	519.17	259.59	15.53	4
Total		8	3342.98			

df - Degree of freedom; Adj SS - Adjusted Sum of Squares.

The contribution of each variable measured as a percentage is shown in Eq. (3) below:

(3) $$\%contribution=\frac{sum\;of\;square\;of\;a\;variable}{total\;sum\;of\;squares}$$

**Figure 5:**
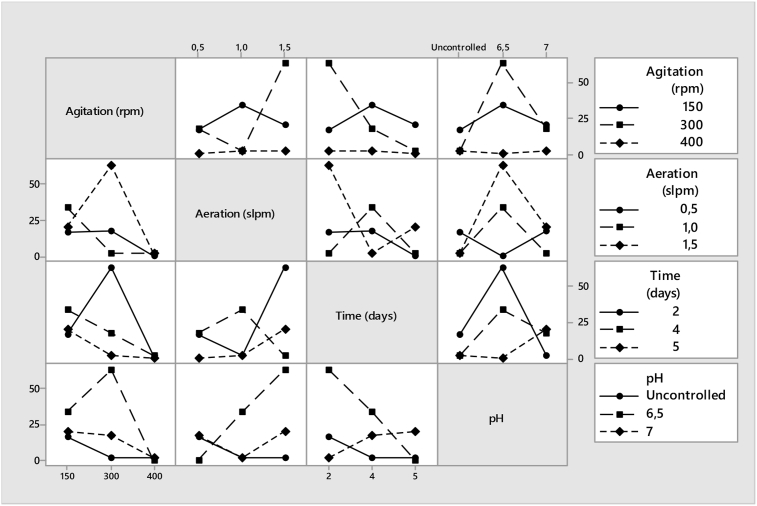
Interaction effect plot of process variables on acetoin concentration (g/L).

Figure 5 shows that the plotted lines were not parallel to each other, indicating the existence of certain relationships between the variables and the acetoin concentration. In Table 6, the percentage contribution of each fermentation variable on acetoin yield was shown in this order: agitation, pH, aeration, and time by 35.52, 31.31, 17.64, and 15.53% respectively.

### 3.7 Performance of the shake flask to the bioreactor study

The optimum conditions established from the acetoin experimental studies using the Taguchi design of mean analysis were agitation of 300 rpm, aeration of 1.5 slpm, day 2 of fermentation, and pH of 6.5 yielding 63.43 g/L of acetoin. The acetoin molar yield of 82.70% was obtained in the bioreactor which is a 3-fold increase (Equation 4) and a corresponding 6-fold increase in concentration compared to our previous study conducted in a shake flask (Table 7). The results also show an improvement compared to previous literature especially for those conducted in a similar reactor system (Table 8). The bioreactor operating conditions used in the work of Xiao et al. (2007) were not optimized. The authors only optimized fermentation media, pretreat molasses with acid, and drops of antifoam were used for hydrolyzation in the bioreactor for 4 h at 60 °C. Though we used a similar strain of *Bacillus* with the authors, the discrepancy reported in the acetoin concentration of the authors compared to our study could be due to changes in the fermentation broth caused by acidification of molasses and unoptimized operating conditions in the bioreactor that might affect the accumulation of acetoin.

**Table 7:** Comparing the shake flask and bioreactor studies performance.

Process Variables	Shake Flask	Bioreactor
Vessel size	250 mL	1.3 L
Agitation	150 rpm	300 rpm
Aeration	-	1.5 slpm
Fermentation time	2 days	2 days
pH	7.0	6.5
Temperature	37 °C	37 °C
Initial glucose concentration	78.40 g/L	156.80 g/L
Acetoin concentration	10.70 g/L	63.43 g/L
Percentage molar yield	28.00%	82.70%

**Table 8:** Biotechnological production of acetoin by different authors compared with present study.

Microorganism	Carbon source	Fermentation variables	Fermentation mode	Manufacturer reactor design specification	Production scale	Maximum concentration	Reference
*Bacillus subtilis* CICC 10025	Molasses	700 rpm, 54.6 h, aeration rate 1vvm, uncontrolled pH, Temp. 37 °C	Batch	BIOSTAT B, B. Braun Biotech International GmbH, Melsungen, Germany	5 L	35.4 g/L	(Xiao et al., 2007)
*Serratia marcescens* H32	Sucrose	600 rpm, 42 h, pH 6.0, aeration rate 1.25 vvm, Temp. 28 °C	Fedbatch	KLF2000 Bioengineering, Wald, Switzerland	3.7 L	60.5 g/L	(Sun et al., 2012)
*Bacillus subtilis* TH-49	Glucose	450 rpm, pH 7.0, aeration rate 1vvm, Temp. 37 °C	Batch	Not specified	100 L	56.9 g/L	(Xu et al., 2011)
*Bacillus subtilis* 168ARSRCPΔacoAΔbdhA strain	Glucose-xylose-arabinose mixture	200 rpm, 120 h, pH 7.0 aeration rate 1vvm, Temp. 37 °C	Fedbatch	New Brunswick Scientific BioFlo 110, USA	1.3 L	62.2 g/L	(Zhang et al., 2016)
*Bacillus amyloliquefaciens* FMME044	Glucose	500 rpm, 48 h, Uncontrolled pH, aeration rate 4 L/min, Temp. 37 °C	Batch	BioFlo 410-7L, New Brunswick Scientific, Enfield, CT, USA	7 L	51.2 g/L	(Zhang et al., 2013)
*Paenibacillus polymyxa* CS107	Glucose	500 rpm, 48 h, pH 6.0, aeration rate 0.5 vvm, Temp. 37 °C	Fedbatch	BIOSTAT B, B. Braun Biotech International GmbH, Melsungen, Germany	5 L	55.3 g/L	(Zhang et al., 2012)
*Bacillus subtilis* SF4-3	Glucose	300 rpm, 96 h, uncontrolled pH, aeration rate 0.5 vvm, Temp. 37 °C	Batch	Shanghai Baoxing Bioengineering Equipment Co., Shanghai, China	5 L	48.9 g/L	(Tian et al., 2016)
*B. subtilis* CICC 10025	Glucose	150 rpm, 2 days, pH 7.0, Temp. 37 °C	Batch	environment-controlled incubator shaker (platform shaker, model: FSIM SP016)	250 mL	10.70 g/L	(Taiwo et al., 2018)
*B. subtilis* CICC 10025	Glucose	300 rpm, 2 days, pH 6.5, aeration rate 1.5 slpm, Temp. 37 °C	Fedbatch	BioFlo/CelliGen 115, New Brunswick Scientific Co., and Eppendorf, Germany	1.3 L	63.43 g/L	**Present study**

∗vvm-volume of air per minute; slpm-standard litre per minute; rpm-revolution per minute. Bold letters in the area of authors reference is to show that our present study and a similar study conducted by our research group on shake flask.

Sun et al. (2012) performed fed-batch fermentation of acetoin using agitation speed control strategy between 500 and 800 rpm. The authors reported that cell growth was favored with higher agitation speeds (700 and 800 rpm). But sucrose consumption rates were relatively lower under these conditions, which lead to lower acetoin production. The author finally used a low agitation speed under fed-batch fermentation resulting in increased acetoin production.

The geometry of bioreactor used by Zhang et al. (2016) was similar to the configuration reported in our present study. The capacity of strain ZB02 used by the authors to produce acetoin was grown in a glucose-xylose-arabinose mixture using fed-batch fermentation. Therefore, increased acetoin reported in their study could be attributed to engineered strain tolerance, the composition of the sugar mixture used as hydrolysate, and the fed-batch fermentation. The authors further stressed the need for optimization.

All other authors (Tian et al., 2016, Xu et al., 2011, Zhang et al., 2012, Zhang et al., 2013) work reported in Table 8 used glucose as their carbon source in acetoin production and chooses their mode of fermentation as either batch or fed-batch. Apart from agitation speed which was chosen based on trial and optimized medium which was emphasized there was no report of other optimized controlled variables used for the different bioreactor specifications. It can be deduced from Table 8, that optimized agitation speed and the choice of right fermentation strategy would enhance acetoin production.

(4) $$\text{Molar}\;\text{yield }\left(\text{\%}\right)\;=\;\frac{\text{g of }{\text{C}}_{4}{\text{H}}_{8}{\text{0}}_{2}\text{ produced × mol.wt of }{\text{C}}_{6}{\text{H}}_{12}{\text{0}}_{6}}{\text{g of }{\text{C}}_{6}{\text{H}}_{12}{\text{0}}_{6}\text{ utilized × mol.wt of }{\text{C}}_{4}{\text{H}}_{8}{\text{0}}_{2}}$$

A six-fold gain (65.43 g/L) of acetoin concentration in the bioreactor studies with 82.70% molar yield was obtained.

## 4 Conclusions

This study provided the statistical optimization technique and fermentation process variables for improved acetoin production from shake flask to batch study in a bioreactor. The statistical model identified the agitation rate as the most controlling variable. It was shown that at the optimum condition, acetoin concentrations of 63.43 g/L with a molar yield of 83% can be achieved in the bioreactor study which was significantly higher when compared to the previously reported study conducted in a shake flask. The results demonstrated that there may be potential for commercial production of acetoin using microbial fermentation with both economic and social benefits.

## Declarations

### Author contribution statement

Abiola Ezekiel Taiwo: Conceived and designed the experiments; Performed the experiments; Analyzed and interpreted the data; Wrote the paper.

Tafirenyika Nyamayaro Madzimbamuto: Conceived and designed the experiments; Analyzed and interpreted the data.

Tunde Victor Ojumu: Conceived and designed the experiments; Analyzed and interpreted the data; Contributed reagents, materials, analysis tools or data; Wrote the paper.

### Funding statement

This work was supported by the 10.13039/501100001321National Research Foundation –10.13039/501100007042Third World Academy of Sciences (NRF-TWAS), grant number (99988 and 116562) for doctoral studies of Abiola Ezekiel Taiwo.

### Competing interest statement

The authors declare no conflict of interest.

### Additional information

No additional information is available for this paper.
